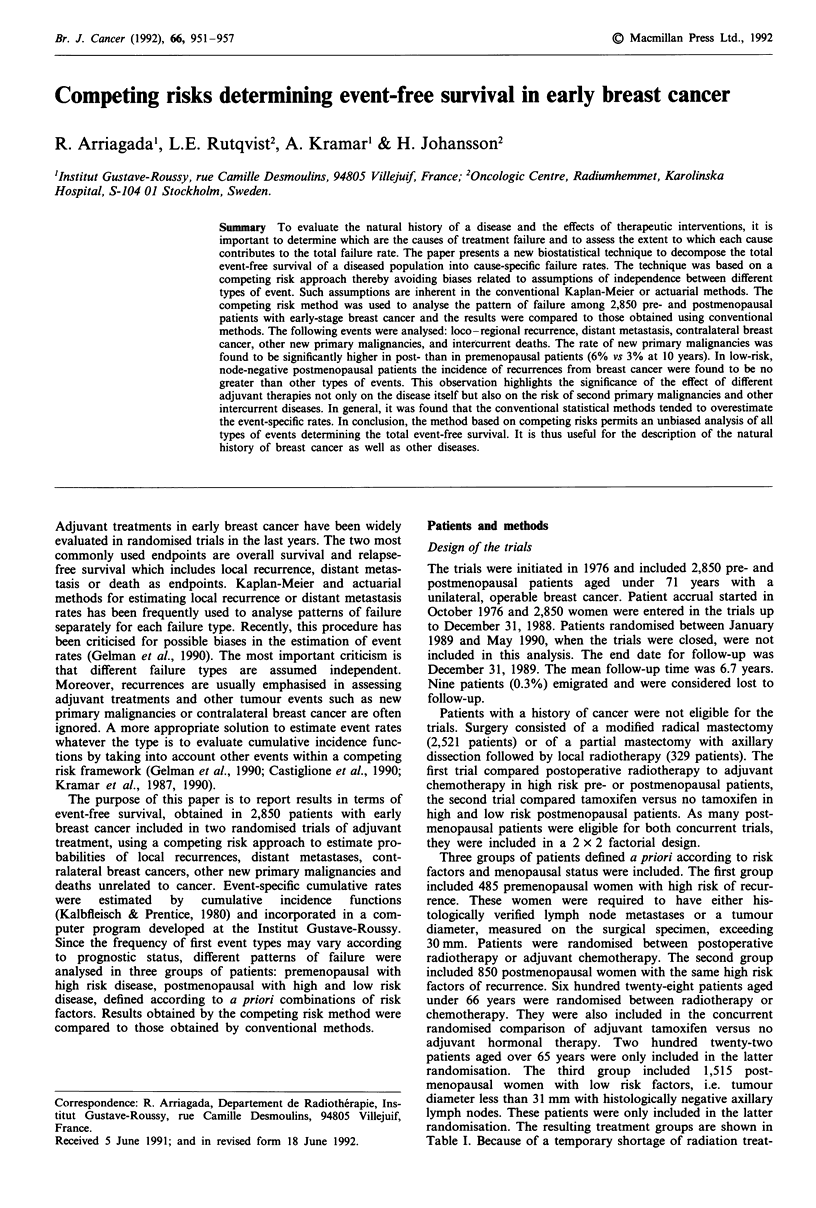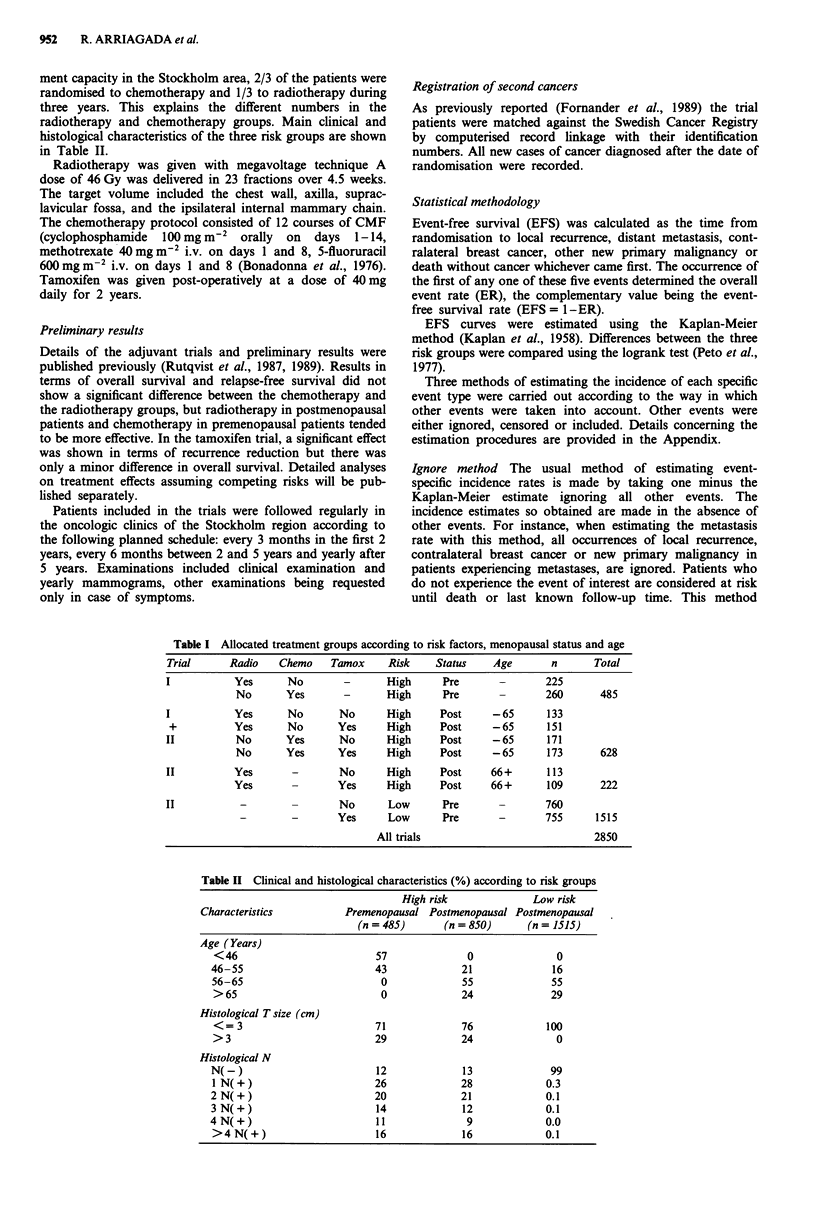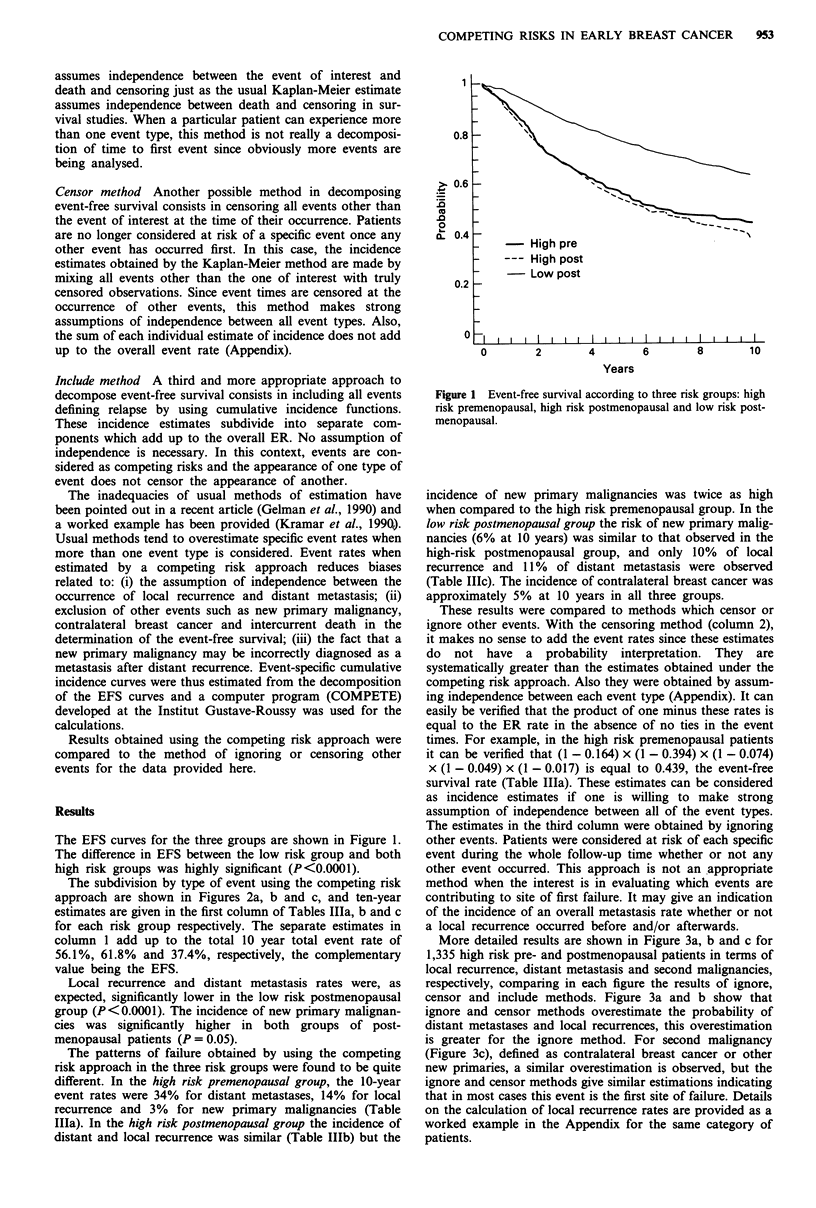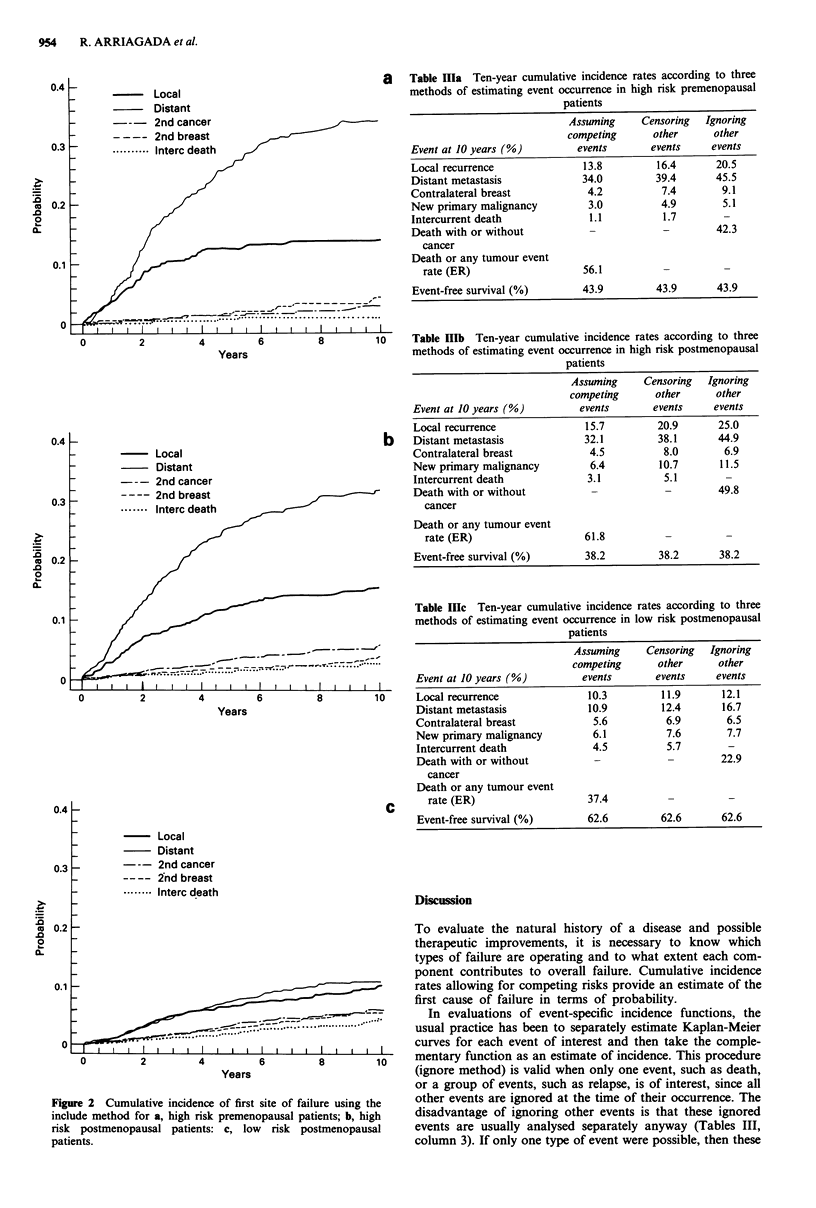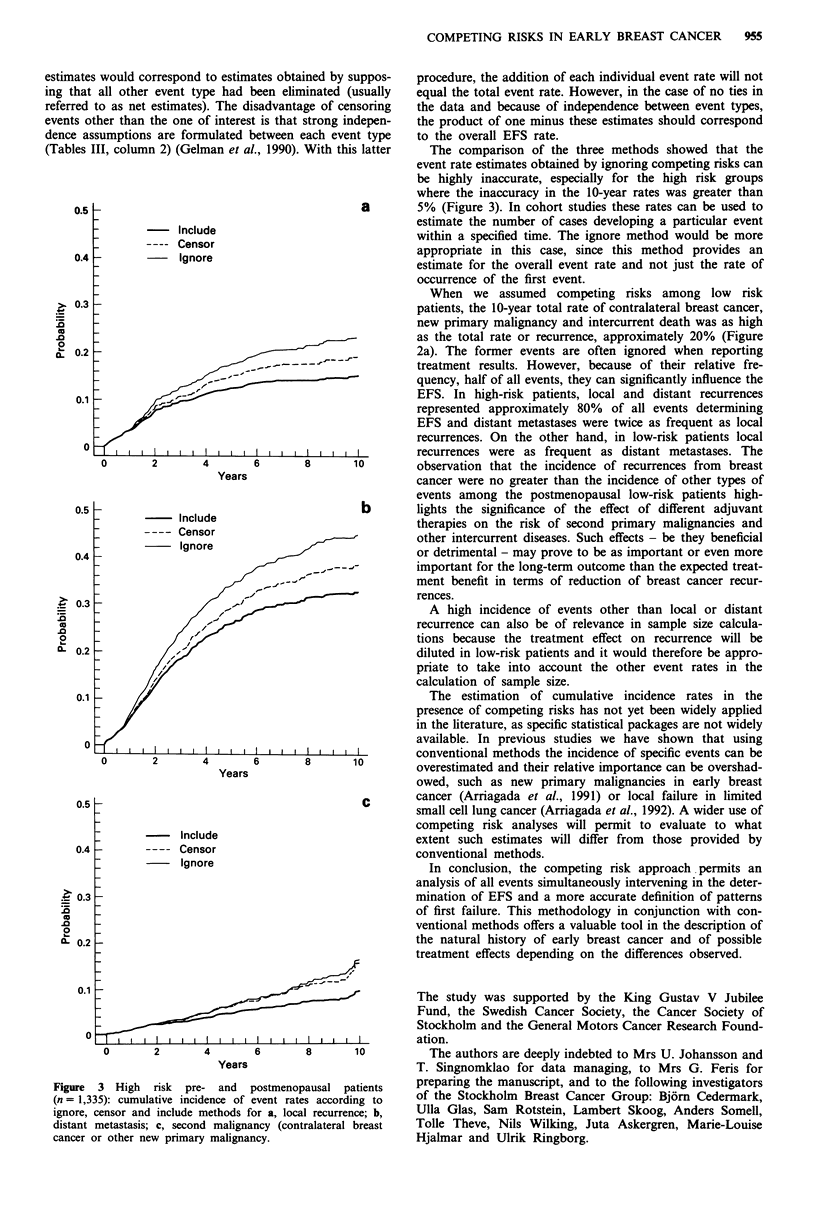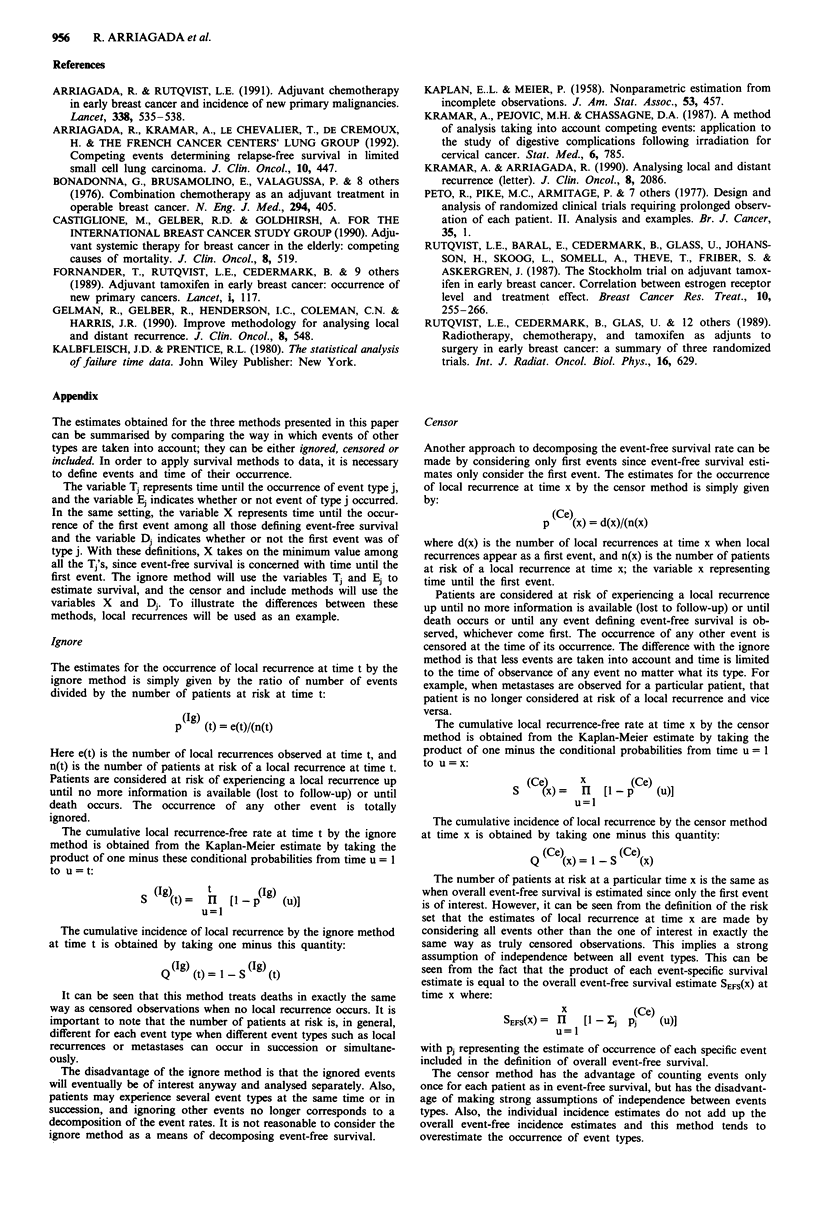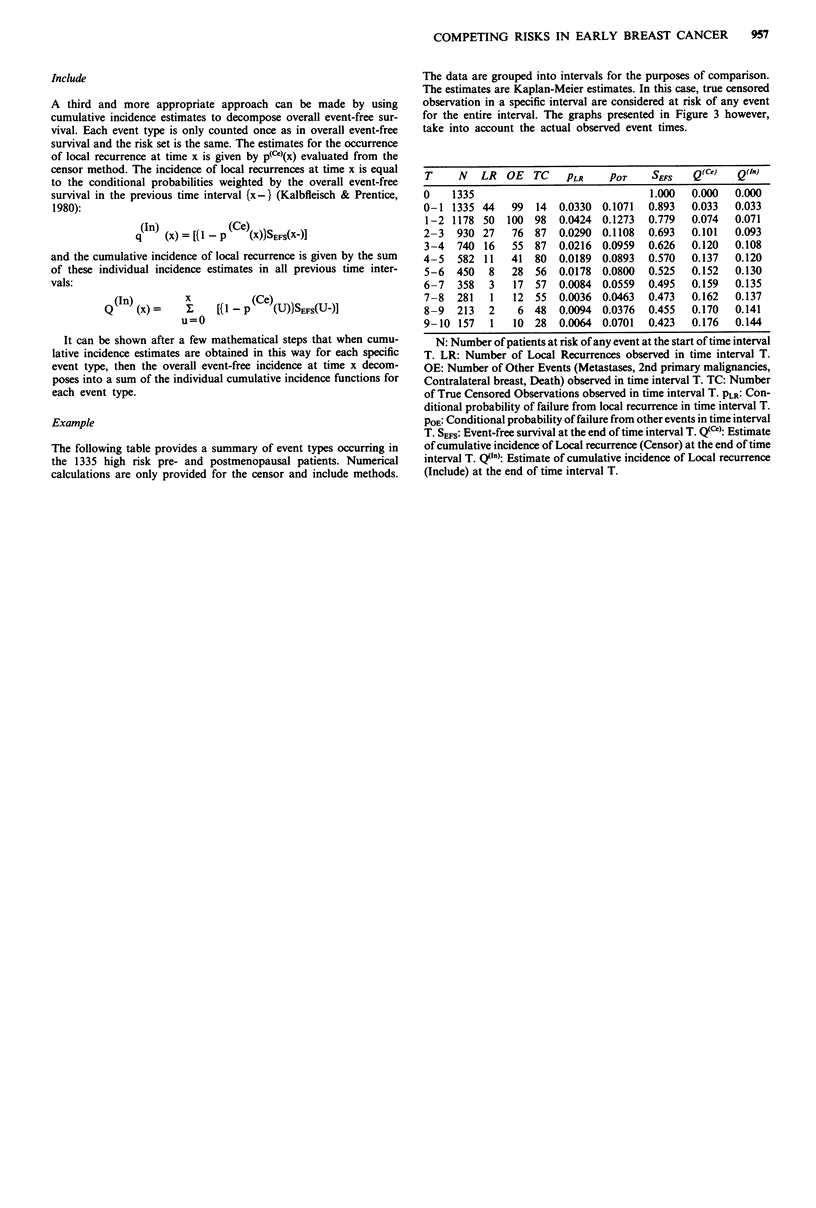# Competing risks determining event-free survival in early breast cancer.

**DOI:** 10.1038/bjc.1992.391

**Published:** 1992-11

**Authors:** R. Arriagada, L. E. Rutqvist, A. Kramar, H. Johansson

**Affiliations:** Institut Gustave-Roussy, Villejuif, France.

## Abstract

To evaluate the natural history of a disease and the effects of therapeutic interventions, it is important to determine which are the causes of treatment failure and to assess the extent to which each cause contributes to the total failure rate. The paper presents a new biostatistical technique to decompose the total event-free survival of a diseased population into cause-specific failure rates. The technique was based on a competing risk approach thereby avoiding biases related to assumptions of independence between different types of event. Such assumptions are inherent in the conventional Kaplan-Meier or actuarial methods. The competing risk method was used to analyse the pattern of failure among 2,850 pre- and postmenopausal patients with early-stage breast cancer and the results were compared to those obtained using conventional methods. The following events were analysed: loco-regional recurrence, distant metastasis, contralateral breast cancer, other new primary malignancies, and intercurrent deaths. The rate of new primary malignancies was found to be significantly higher in post- than in premenopausal patients (6% vs 3% at 10 years). In low-risk, node-negative postmenopausal patients the incidence of recurrences from breast cancer were found to be no greater than other types of events. This observation highlights the significance of the effect of different adjuvant therapies not only on the disease itself but also on the risk of second primary malignancies and other intercurrent diseases. In general, it was found that the conventional statistical methods tended to overestimate the event-specific rates. In conclusion, the method based on competing risks permits an unbiased analysis of all types of events determining the total event-free survival. It is thus useful for the description of the natural history of breast cancer as well as other diseases.


*Br. J. Cancer* (1992), **66**, 951–957 © Macmillan Press Ltd., 1992

### Competing risks determining event-free survival in early breast cancer

R. Arriagada[1], L.E. Rutqvist[2], A. Kramar[1] & H. Johansson[2]

[1]*Institut Gustave-Roussy, rue Camille Desmoulins, 94805 Villejuif, France;* [2]*Oncologic Centre, Radiumhemmet, Karolinska Hospital, S-104 01 Stockholm, Sweden.*

**Summary** To evaluate the natural history of a disease and the effects of therapeutic interventions, it is important to determine which are the causes of treatment failure and to assess the extent to which each cause contributes to the total failure rate. The paper presents a new biostatistical technique to decompose the total event-free survival of a diseased population into cause-specific failure rates. The technique was based on a competing risk approach thereby avoiding biases related to assumptions of independence between different types of event. Such assumptions are inherent in the conventional Kaplan-Meier or actuarial methods. The competing risk method was used to analyse the pattern of failure among 2,850 pre- and postmenopausal patients with early-stage breast cancer and the results were compared to those obtained using conventional methods. The following events were analysed: loco-regional recurrence, distant metastasis, contralateral breast cancer, other new primary malignancies, and intercurrent deaths. The rate of new primary malignancies was found to be significantly higher in post- than in premenopausal patients (6% *vs* 3% at 10 years). In low-risk, node-negative postmenopausal patients the incidence of recurrences from breast cancer were found to be no greater than other types of events. This observation highlights the significance of the effect of different adjuvant therapies not only on the disease itself but also on the risk of second primary malignancies and other intercurrent diseases. In general, it was found that the conventional statistical methods tended to overestimate the event-specific rates. In conclusion, the method based on competing risks permits an unbiased analysis of all types of events determining the total event-free survival. It is thus useful for the description of the natural history of breast cancer as well as other diseases.

Adjuvant treatments in early breast cancer have been widely evaluated in randomised trials in the last years. The two most commonly used endpoints are overall survival and relapse-free survival which includes local recurrence, distant metastasis or death as endpoints. Kaplan-Meier and actuarial methods for estimating local recurrence or distant metastasis rates has been frequently used to analyse patterns of failure separately for each failure type. Recently, this procedure has been criticised for possible biases in the estimation of event rates (Gelman *et al.*, 1990). The most important criticism is that different failure types are assumed independent. Moreover, recurrences are usually emphasised in assessing adjuvant treatments and other tumour events such as new primary malignancies or contralateral breast cancer are often ignored. A more appropriate solution to estimate event rates whatever the type is to evaluate cumulative incidence functions by taking into account other events within a competing risk framework (Gelman *et al.*, 1990; Castiglione *et al.*, 1990; Kramar *et al.*, 1987, 1990).

The purpose of this paper is to report results in terms of event-free survival, obtained in 2,850 patients with early breast cancer included in two randomised trials of adjuvant treatment, using a competing risk approach to estimate probabilities of local recurrences, distant metastases, contralateral breast cancers, other new primary malignancies and deaths unrelated to cancer. Event-specific cumulative rates were estimated by cumulative incidence functions (Kalbfleisch & Prentice, 1980) and incorporated in a computer program developed at the Institut Gustave-Roussy. Since the frequency of first event types may vary according to prognostic status, different patterns of failure were analysed in three groups of patients: premenopausal with high risk disease, postmenopausal with high and low risk disease, defined according to *a priori* combinations of risk factors. Results obtained by the competing risk method were compared to those obtained by conventional methods.

Correspondence: R. Arriagada, Departement de Radiothérapie, Institut Gustave-Roussy, rue Camille Desmoulins, 94805 Villejuif, France.
Received 5 June 1991; and in revised form 18 June 1992.

### Patients and methods

### *Design of the trials*

The trials were initiated in 1976 and included 2,850 pre- and postmenopausal patients aged under 71 years with a unilateral, operable breast cancer. Patient accrual started in October 1976 and 2,850 women were entered in the trials up to December 31, 1988. Patients randomised between January 1989 and May 1990, when the trials were closed, were not included in this analysis. The end date for follow-up was December 31, 1989. The mean follow-up time was 6.7 years. Nine patients (0.3%) emigrated and were considered lost to follow-up.

Patients with a history of cancer were not eligible for the trials. Surgery consisted of a modified radical mastectomy (2,521 patients) or of a partial mastectomy with axillary dissection followed by local radiotherapy (329 patients). The first trial compared postoperative radiotherapy to adjuvant chemotherapy in high risk pre- or postmenopausal patients, the second trial compared tamoxifen versus no tamoxifen in high and low risk postmenopausal patients. As many postmenopausal patients were eligible for both concurrent trials, they were included in a 2 × 2 factorial design.

Three groups of patients defined *a priori* according to risk factors and menopausal status were included. The first group included 485 premenopausal women with high risk of recurrence. These women were required to have either histologically verified lymph node metastases or a tumour diameter, measured on the surgical specimen, exceeding 30 mm. Patients were randomised between postoperative radiotherapy or adjuvant chemotherapy. The second group included 850 postmenopausal women with the same high risk factors of recurrence. Six hundred twenty-eight patients aged under 66 years were randomised between radiotherapy or chemotherapy. They were also included in the concurrent randomised comparison of adjuvant tamoxifen versus no adjuvant hormonal therapy. Two hundred twenty-two patients aged over 65 years were only included in the latter randomisation. The third group included 1,515 postmenopausal women with low risk factors, i.e. tumour diameter less than 31 mm with histologically negative axillary lymph nodes. These patients were only included in the latter randomisation. The resulting treatment groups are shown in Table I. Because of a temporary shortage of radiation treatment capacity in the Stockholm area, 2/3 of the patients were randomised to chemotherapy and 1/3 to radiotherapy during three years. This explains the different numbers in the radiotherapy and chemotherapy groups. Main clinical and histological characteristics of the three risk groups are shown in Table II.

Radiotherapy was given with megavoltage technique A dose of 46 Gy was delivered in 23 fractions over 4.5 weeks. The target volume included the chest wall, axilla, supraclavicular fossa, and the ipsilateral internal mammary chain. The chemotherapy protocol consisted of 12 courses of CMF (cyclophosphamide 100 mg $m^{-2}$ orally on days 1–14, methotrexate 40 mg $m^{-2}$ i.v. on days 1 and 8, 5-fluoruracil 600 mg $m^{-2}$ i.v. on days 1 and 8 (Bonadonna *et al.*, 1976). Tamoxifen was given post-operatively at a dose of 40 mg daily for 2 years.

### *Preliminary results*

Details of the adjuvant trials and preliminary results were published previously (Rutqvist *et al.*, 1987, 1989). Results in terms of overall survival and relapse-free survival did not show a significant difference between the chemotherapy and the radiotherapy groups, but radiotherapy in postmenopausal patients and chemotherapy in premenopausal patients tended to be more effective. In the tamoxifen trial, a significant effect was shown in terms of recurrence reduction but there was only a minor difference in overall survival. Detailed analyses on treatment effects assuming competing risks will be published separately.

Patients included in the trials were followed regularly in the oncologic clinics of the Stockholm region according to the following planned schedule: every 3 months in the first 2 years, every 6 months between 2 and 5 years and yearly after 5 years. Examinations included clinical examination and yearly mammograms, other examinations being requested only in case of symptoms.

### *Registration of second cancers*

As previously reported (Fornander *et al.*, 1989) the trial patients were matched against the Swedish Cancer Registry by computerised record linkage with their identification numbers. All new cases of cancer diagnosed after the date of randomisation were recorded.

### *Statistical methodology*

Event-free survival (EFS) was calculated as the time from randomisation to local recurrence, distant metastasis, contralateral breast cancer, other new primary malignancy or death without cancer whichever came first. The occurrence of the first of any one of these five events determined the overall event rate (ER), the complementary value being the event-free survival rate (EFS = 1 – ER).

EFS curves were estimated using the Kaplan-Meier method (Kaplan *et al.*, 1958). Differences between the three risk groups were compared using the logrank test (Peto *et al.*, 1977).

Three methods of estimating the incidence of each specific event type were carried out according to the way in which other events were taken into account. Other events were either ignored, censored or included. Details concerning the estimation procedures are provided in the Appendix.

*Ignore method* The usual method of estimating event-specific incidence rates is made by taking one minus the Kaplan-Meier estimate ignoring all other events. The incidence estimates so obtained are made in the absence of other events. For instance, when estimating the metastasis rate with this method, all occurrences of local recurrence, contralateral breast cancer or new primary malignancy in patients experiencing metastases, are ignored. Patients who do not experience the event of interest are considered at risk until death or last known follow-up time. This method

**Table I** Allocated treatment groups according to risk factors, menopausal status and age

| *Trial* | *Radio* | *Chemo* | *Tamox* | *Risk* | *Status* | *Age* | *n* | *Total* |
|---|---|---|---|---|---|---|---|---|
| I | Yes | No | – | High | Pre | – | 225 | |
| | No | Yes | – | High | Pre | – | 260 | 485 |
| I | Yes | No | No | High | Post | –65 | 133 | |
| + | Yes | No | Yes | High | Post | –65 | 151 | |
| II | No | Yes | No | High | Post | –65 | 171 | |
| | No | Yes | Yes | High | Post | –65 | 173 | 628 |
| II | Yes | – | No | High | Post | 66+ | 113 | |
| | Yes | – | Yes | High | Post | 66+ | 109 | 222 |
| II | – | – | No | Low | Pre | – | 760 | |
| | – | – | Yes | Low | Pre | – | 755 | 1515 |
| | | | | All trials | | | | 2850 |

**Table II** Clinical and histological characteristics (%) according to risk groups

| *Characteristics* | *High risk* Premenopausal (n = 485) | *High risk* Postmenopausal (n = 850) | *Low risk* Postmenopausal (n = 1515) |
|---|---|---|---|
| *Age (Years)* | | | |
| <46 | 57 | 0 | 0 |
| 46–55 | 43 | 21 | 16 |
| 56–65 | 0 | 55 | 55 |
| >65 | 0 | 24 | 29 |
| *Histological T size (cm)* | | | |
| <= 3 | 71 | 76 | 100 |
| >3 | 29 | 24 | 0 |
| *Histological N* | | | |
| N(–) | 12 | 13 | 99 |
| 1 N(+) | 26 | 28 | 0.3 |
| 2 N(+) | 20 | 21 | 0.1 |
| 3 N(+) | 14 | 12 | 0.1 |
| 4 N(+) | 11 | 9 | 0.0 |
| >4 N(+) | 16 | 16 | 0.1 |

assumes independence between the event of interest and death and censoring just as the usual Kaplan-Meier estimate assumes independence between death and censoring in survival studies. When a particular patient can experience more than one event type, this method is not really a decomposition of time to first event since obviously more events are being analysed.

*Censor method* Another possible method in decomposing event-free survival consists in censoring all events other than the event of interest at the time of their occurrence. Patients are no longer considered at risk of a specific event once any other event has occurred first. In this case, the incidence estimates obtained by the Kaplan-Meier method are made by mixing all events other than the one of interest with truly censored observations. Since event times are censored at the occurrence of other events, this method makes strong assumptions of independence between all event types. Also, the sum of each individual estimate of incidence does not add up to the overall event rate (Appendix).

*Include method* A third and more appropriate approach to decompose event-free survival consists in including all events defining relapse by using cumulative incidence functions. These incidence estimates subdivide into separate components which add up to the overall ER. No assumption of independence is necessary. In this context, events are considered as competing risks and the appearance of one type of event does not censor the appearance of another.

The inadequacies of usual methods of estimation have been pointed out in a recent article (Gelman *et al.*, 1990) and a worked example has been provided (Kramar *et al.*, 1990). Usual methods tend to overestimate specific event rates when more than one event type is considered. Event rates when estimated by a competing risk approach reduces biases related to: (i) the assumption of independence between the occurrence of local recurrence and distant metastasis; (ii) exclusion of other events such as new primary malignancy, contralateral breast cancer and intercurrent death in the determination of the event-free survival; (iii) the fact that a new primary malignancy may be incorrectly diagnosed as a metastasis after distant recurrence. Event-specific cumulative incidence curves were thus estimated from the decomposition of the EFS curves and a computer program (COMPETE) developed at the Institut Gustave-Roussy was used for the calculations.

Results obtained using the competing risk approach were compared to the method of ignoring or censoring other events for the data provided here.

**Figure 1** Event-free survival according to three risk groups: high risk premenopausal, high risk postmenopausal and low risk postmenopausal.

### Results

The EFS curves for the three groups are shown in Figure 1. The difference in EFS between the low risk group and both high risk groups was highly significant ($P<0.0001$).

The subdivision by type of event using the competing risk approach are shown in Figures 2a, b and c, and ten-year estimates are given in the first column of Tables IIIa, b and c for each risk group respectively. The separate estimates in column 1 add up to the total 10 year total event rate of 56.1%, 61.8% and 37.4%, respectively, the complementary value being the EFS.

Local recurrence and distant metastasis rates were, as expected, significantly lower in the low risk postmenopausal group ($P<0.0001$). The incidence of new primary malignancies was significantly higher in both groups of postmenopausal patients ($P = 0.05$).

The patterns of failure obtained by using the competing risk approach in the three risk groups were found to be quite different. In the *high risk premenopausal group*, the 10-year event rates were 34% for distant metastases, 14% for local recurrence and 3% for new primary malignancies (Table IIIa). In the *high risk postmenopausal group* the incidence of distant and local recurrence was similar (Table IIIb) but the incidence of new primary malignancies was twice as high when compared to the high risk premenopausal group. In the *low risk postmenopausal group* the risk of new primary malignancies (6% at 10 years) was similar to that observed in the high-risk postmenopausal group, and only 10% of local recurrence and 11% of distant metastasis were observed (Table IIIc). The incidence of contralateral breast cancer was approximately 5% at 10 years in all three groups.

These results were compared to methods which censor or ignore other events. With the censoring method (column 2), it makes no sense to add the event rates since these estimates do not have a probability interpretation. They are systematically greater than the estimates obtained under the competing risk approach. Also they were obtained by assuming independence between each event type (Appendix). It can easily be verified that the product of one minus these rates is equal to the ER rate in the absence of no ties in the event times. For example, in the high risk premenopausal patients it can be verified that $(1 - 0.164) \times (1 - 0.394) \times (1 - 0.074) \times (1 - 0.049) \times (1 - 0.017)$ is equal to 0.439, the event-free survival rate (Table IIIa). These estimates can be considered as incidence estimates if one is willing to make strong assumption of independence between all of the event types. The estimates in the third column were obtained by ignoring other events. Patients were considered at risk of each specific event during the whole follow-up time whether or not any other event occurred. This approach is not an appropriate method when the interest is in evaluating which events are contributing to site of first failure. It may give an indication of the incidence of an overall metastasis rate whether or not a local recurrence occurred before and/or afterwards.

More detailed results are shown in Figure 3a, b and c for 1,335 high risk pre- and postmenopausal patients in terms of local recurrence, distant metastasis and second malignancies, respectively, comparing in each figure the results of ignore, censor and include methods. Figure 3a and b show that ignore and censor methods overestimate the probability of distant metastases and local recurrences, this overestimation is greater for the ignore method. For second malignancy (Figure 3c), defined as contralateral breast cancer or other new primaries, a similar overestimation is observed, but the ignore and censor methods give similar estimations indicating that in most cases this event is the first site of failure. Details on the calculation of local recurrence rates are provided as a worked example in the Appendix for the same category of patients.

**Figure 2** Cumulative incidence of first site of failure using the include method for **a**, high risk premenopausal patients; **b**, high risk postmenopausal patients: **c**, low risk postmenopausal patients.

**Table IIIa** Ten-year cumulative incidence rates according to three methods of estimating event occurrence in high risk premenopausal patients

| *Event at 10 years (%)* | *Assuming competing events* | *Censoring other events* | *Ignoring other events* |
|---|---|---|---|
| Local recurrence | 13.8 | 16.4 | 20.5 |
| Distant metastasis | 34.0 | 39.4 | 45.5 |
| Contralateral breast | 4.2 | 7.4 | 9.1 |
| New primary malignancy | 3.0 | 4.9 | 5.1 |
| Intercurrent death | 1.1 | 1.7 | – |
| Death with or without cancer | – | – | 42.3 |
| Death or any tumour event rate (ER) | 56.1 | – | – |
| Event-free survival (%) | 43.9 | 43.9 | 43.9 |

**Table IIIb** Ten-year cumulative incidence rates according to three methods of estimating event occurrence in high risk postmenopausal patients

| *Event at 10 years (%)* | *Assuming competing events* | *Censoring other events* | *Ignoring other events* |
|---|---|---|---|
| Local recurrence | 15.7 | 20.9 | 25.0 |
| Distant metastasis | 32.1 | 38.1 | 44.9 |
| Contralateral breast | 4.5 | 8.0 | 6.9 |
| New primary malignancy | 6.4 | 10.7 | 11.5 |
| Intercurrent death | 3.1 | 5.1 | – |
| Death with or without cancer | – | – | 49.8 |
| Death or any tumour event rate (ER) | 61.8 | – | – |
| Event-free survival (%) | 38.2 | 38.2 | 38.2 |

**Table IIIc** Ten-year cumulative incidence rates according to three methods of estimating event occurrence in low risk postmenopausal patients

| *Event at 10 years (%)* | *Assuming competing events* | *Censoring other events* | *Ignoring other events* |
|---|---|---|---|
| Local recurrence | 10.3 | 11.9 | 12.1 |
| Distant metastasis | 10.9 | 12.4 | 16.7 |
| Contralateral breast | 5.6 | 6.9 | 6.5 |
| New primary malignancy | 6.1 | 7.6 | 7.7 |
| Intercurrent death | 4.5 | 5.7 | – |
| Death with or without cancer | – | – | 22.9 |
| Death or any tumour event rate (ER) | 37.4 | – | – |
| Event-free survival (%) | 62.6 | 62.6 | 62.6 |

### Discussion

To evaluate the natural history of a disease and possible therapeutic improvements, it is necessary to know which types of failure are operating and to what extent each component contributes to overall failure. Cumulative incidence rates allowing for competing risks provide an estimate of the first cause of failure in terms of probability.

In evaluations of event-specific incidence functions, the usual practice has been to separately estimate Kaplan-Meier curves for each event of interest and then take the complementary function as an estimate of incidence. This procedure (ignore method) is valid when only one event, such as death, or a group of events, such as relapse, is of interest, since all other events are ignored at the time of their occurrence. The disadvantage of ignoring other events is that these ignored events are usually analysed separately anyway (Tables III, column 3). If only one type of event were possible, then these estimates would correspond to estimates obtained by supposing that all other event type had been eliminated (usually referred to as net estimates). The disadvantage of censoring events other than the one of interest is that strong independence assumptions are formulated between each event type (Tables III, column 2) (Gelman *et al.*, 1990). With this latter procedure, the addition of each individual event rate will not equal the total event rate. However, in the case of no ties in the data and because of independence between event types, the product of one minus these estimates should correspond to the overall EFS rate.

**Figure 3** High risk pre- and postmenopausal patients ($n = 1,335$): cumulative incidence of event rates according to ignore, censor and include methods for **a**, local recurrence; **b**, distant metastasis; **c**, second malignancy (contralateral breast cancer or other new primary malignancy.

The comparison of the three methods showed that the event rate estimates obtained by ignoring competing risks can be highly inaccurate, especially for the high risk groups where the inaccuracy in the 10-year rates was greater than 5% (Figure 3). In cohort studies these rates can be used to estimate the number of cases developing a particular event within a specified time. The ignore method would be more appropriate in this case, since this method provides an estimate for the overall event rate and not just the rate of occurrence of the first event.

When we assumed competing risks among low risk patients, the 10-year total rate of contralateral breast cancer, new primary malignancy and intercurrent death was as high as the total rate or recurrence, approximately 20% (Figure 2a). The former events are often ignored when reporting treatment results. However, because of their relative frequency, half of all events, they can significantly influence the EFS. In high-risk patients, local and distant recurrences represented approximately 80% of all events determining EFS and distant metastases were twice as frequent as local recurrences. On the other hand, in low-risk patients local recurrences were as frequent as distant metastases. The observation that the incidence of recurrences from breast cancer were no greater than the incidence of other types of events among the postmenopausal low-risk patients highlights the significance of the effect of different adjuvant therapies on the risk of second primary malignancies and other intercurrent diseases. Such effects – be they beneficial or detrimental – may prove to be as important or even more important for the long-term outcome than the expected treatment benefit in terms of reduction of breast cancer recurrences.

A high incidence of events other than local or distant recurrence can also be of relevance in sample size calculations because the treatment effect on recurrence will be diluted in low-risk patients and it would therefore be appropriate to take into account the other event rates in the calculation of sample size.

The estimation of cumulative incidence rates in the presence of competing risks has not yet been widely applied in the literature, as specific statistical packages are not widely available. In previous studies we have shown that using conventional methods the incidence of specific events can be overestimated and their relative importance can be overshadowed, such as new primary malignancies in early breast cancer (Arriagada *et al.*, 1991) or local failure in limited small cell lung cancer (Arriagada *et al.*, 1992). A wider use of competing risk analyses will permit to evaluate to what extent such estimates will differ from those provided by conventional methods.

In conclusion, the competing risk approach permits an analysis of all events simultaneously intervening in the determination of EFS and a more accurate definition of patterns of first failure. This methodology in conjunction with conventional methods offers a valuable tool in the description of the natural history of early breast cancer and of possible treatment effects depending on the differences observed.

The study was supported by the King Gustav V Jubilee Fund, the Swedish Cancer Society, the Cancer Society of Stockholm and the General Motors Cancer Research Foundation.

The authors are deeply indebted to Mrs U. Johansson and T. Singnomklao for data managing, to Mrs G. Feris for preparing the manuscript, and to the following investigators of the Stockholm Breast Cancer Group: Björn Cedermark, Ulla Glas, Sam Rotstein, Lambert Skoog, Anders Somell, Tolle Theve, Nils Wilking, Juta Askergren, Marie-Louise Hjalmar and Ulrik Ringborg.

### References

ARRIAGADA, R. & RUTQVIST, L.E. (1991). Adjuvant chemotherapy in early breast cancer and incidence of new primary malignancies. *Lancet*, **338**, 535–538.

ARRIAGADA, R., KRAMAR, A., LE CHEVALIER, T., DE CREMOUX, H. & THE FRENCH CANCER CENTERS' LUNG GROUP (1992). Competing events determining relapse-free survival in limited small cell lung carcinoma. *J. Clin. Oncol.*, **10**, 447.

BONADONNA, G., BRUSAMOLINO, E., VALAGUSSA, P. & 8 others (1976). Combination chemotherapy as an adjuvant treatment in operable breast cancer. *N. Eng. J. Med.*, **294**, 405.

CASTIGLIONE, M., GELBER, R.D. & GOLDHIRSH, A. FOR THE INTERNATIONAL BREAST CANCER STUDY GROUP (1990). Adjuvant systemic therapy for breast cancer in the elderly: competing causes of mortality. *J. Clin. Oncol.*, **8**, 519.

FORNANDER, T., RUTQVIST, L.E., CEDERMARK, B. & 9 others (1989). Adjuvant tamoxifen in early breast cancer: occurrence of new primary cancers. *Lancet*, **i**, 117.

GELMAN, R., GELBER, R., HENDERSON, I.C., COLEMAN, C.N. & HARRIS, J.R. (1990). Improve methodology for analysing local and distant recurrence. *J. Clin. Oncol.*, **8**, 548.

KALBFLEISCH, J.D. & PRENTICE, R.L. (1980). *The statistical analysis of failure time data.* John Wiley Publisher: New York.

KAPLAN, E..L. & MEIER, P. (1958). Nonparametric estimation from incomplete observations. *J. Am. Stat. Assoc.*, **53**, 457.

KRAMAR, A., PEJOVIC, M.H. & CHASSAGNE, D.A. (1987). A method of analysis taking into account competing events: application to the study of digestive complications following irradiation for cervical cancer. *Stat. Med.*, **6**, 785.

KRAMAR, A. & ARRIAGADA, R. (1990). Analysing local and distant recurrence (letter). *J. Clin. Oncol.*, **8**, 2086.

PETO, R., PIKE, M.C., ARMITAGE, P. & 7 others (1977). Design and analysis of randomized clinical trials requiring prolonged observation of each patient. II. Analysis and examples. *Br. J. Cancer*, **35**, 1.

RUTQVIST, L.E., BARAL, E., CEDERMARK, B., GLASS, U., JOHANSSON, H., SKOOG, L., SOMELL, A., THEVE, T., FRIBER, S. & ASKERGREN, J. (1987). The Stockholm trial on adjuvant tamoxifen in early breast cancer. Correlation between estrogen receptor level and treatment effect. *Breast Cancer Res. Treat.*, **10**, 255–266.

RUTQVIST, L.E., CEDERMARK, B., GLAS, U. & 12 others (1989). Radiotherapy, chemotherapy, and tamoxifen as adjunts to surgery in early breast cancer: a summary of three randomized trials. *Int. J. Radiat. Oncol. Biol. Phys.*, **16**, 629.

### Appendix

The estimates obtained for the three methods presented in this paper can be summarised by comparing the way in which events of other types are taken into account; they can be either *ignored, censored or included.* In order to apply survival methods to data, it is necessary to define events and time of their occurrence.

The variable $T_j$ represents time until the occurrence of event type j, and the variable $E_j$ indicates whether or not event of type j occurred. In the same setting, the variable X represents time until the occurrence of the first event among all those defining event-free survival and the variable $D_j$ indicates whether or not the first event was of type j. With these definitions, X takes on the minimum value among all the $T_j$'s, since event-free survival is concerned with time until the first event. The ignore method will use the variables $T_j$ and $E_j$ to estimate survival, and the censor and include methods will use the variables X and $D_j$. To illustrate the differences between these methods, local recurrences will be used as an example.

*Ignore*

The estimates for the occurrence of local recurrence at time t by the ignore method is simply given by the ratio of number of events divided by the number of patients at risk at time t:

$$p^{(Ig)}(t) = e(t)/(n(t)$$

Here e(t) is the number of local recurrences observed at time t, and n(t) is the number of patients at risk of a local recurrence at time t. Patients are considered at risk of experiencing a local recurrence up until no more information is available (lost to follow-up) or until death occurs. The occurrence of any other event is totally ignored.

The cumulative local recurrence-free rate at time t by the ignore method is obtained from the Kaplan-Meier estimate by taking the product of one minus these conditional probabilities from time u = 1 to u = t:

$$S^{(Ig)}(t) = \prod_{u=1}^{t} [1 - p^{(Ig)}(u)]$$

The cumulative incidence of local recurrence by the ignore method at time t is obtained by taking one minus this quantity:

$$Q^{(Ig)}(t) = 1 - S^{(Ig)}(t)$$

It can be seen that this method treats deaths in exactly the same way as censored observations when no local recurrence occurs. It is important to note that the number of patients at risk is, in general, different for each event type when different event types such as local recurrences or metastases can occur in succession or simultaneously.

The disadvantage of the ignore method is that the ignored events will eventually be of interest anyway and analysed separately. Also, patients may experience several event types at the same time or in succession, and ignoring other events no longer corresponds to a decomposition of the event rates. It is not reasonable to consider the ignore method as a means of decomposing event-free survival.

*Censor*

Another approach to decomposing the event-free survival rate can be made by considering only first events since event-free survival estimates only consider the first event. The estimates for the occurrence of local recurrence at time x by the censor method is simply given by:

$$p^{(Ce)}(x) = d(x)/(n(x)$$

where d(x) is the number of local recurrences at time x when local recurrences appear as a first event, and n(x) is the number of patients at risk of a local recurrence at time x; the variable x representing time until the first event.

Patients are considered at risk of experiencing a local recurrence up until no more information is available (lost to follow-up) or until death occurs or until any event defining event-free survival is observed, whichever come first. The occurrence of any other event is censored at the time of its occurrence. The difference with the ignore method is that less events are taken into account and time is limited to the time of observance of any event no matter what its type. For example, when metastases are observed for a particular patient, that patient is no longer considered at risk of a local recurrence and vice versa.

The cumulative local recurrence-free rate at time x by the censor method is obtained from the Kaplan-Meier estimate by taking the product of one minus the conditional probabilities from time u = 1 to u = x:

$$S^{(Ce)}(x) = \prod_{u=1}^{x} [1 - p^{(Ce)}(u)]$$

The cumulative incidence of local recurrence by the censor method at time x is obtained by taking one minus this quantity:

$$Q^{(Ce)}(x) = 1 - S^{(Ce)}(x)$$

The number of patients at risk at a particular time x is the same as when overall event-free survival is estimated since only the first event is of interest. However, it can be seen from the definition of the risk set that the estimates of local recurrence at time x are made by considering all events other than the one of interest in exactly the same way as truly censored observations. This implies a strong assumption of independence between all event types. This can be seen from the fact that the product of each event-specific survival estimate is equal to the overall event-free survival estimate $S_{EFS}(x)$ at time x where:

$$S_{EFS}(x) = \prod_{u=1}^{x} [1 - \Sigma_j \; p_j^{(Ce)}(u)]$$

with $p_j$ representing the estimate of occurrence of each specific event included in the definition of overall event-free survival.

The censor method has the advantage of counting events only once for each patient as in event-free survival, but has the disadvantage of making strong assumptions of independence between events types. Also, the individual incidence estimates do not add up the overall event-free incidence estimates and this method tends to overestimate the occurrence of event types.

*Include*

A third and more appropriate approach can be made by using cumulative incidence estimates to decompose overall event-free survival. Each event type is only counted once as in overall event-free survival and the risk set is the same. The estimates for the occurrence of local recurrence at time x is given by $p^{(Ce)}(x)$ evaluated from the censor method. The incidence of local recurrences at time x is equal to the conditional probabilities weighted by the overall event-free survival in the previous time interval {x−} (Kalbfleisch & Prentice, 1980):

$$q^{(In)}(x) = [\{1 - p^{(Ce)}(x)\}S_{EFS}(x-)]$$

and the cumulative incidence of local recurrence is given by the sum of these individual incidence estimates in all previous time intervals:

$$Q^{(In)}(x) = \sum_{u=0}^{x} [\{1 - p^{(Ce)}(U)\}S_{EFS}(U-)]$$

It can be shown after a few mathematical steps that when cumulative incidence estimates are obtained in this way for each specific event type, then the overall event-free incidence at time x decomposes into a sum of the individual cumulative incidence functions for each event type.

*Example*

The following table provides a summary of event types occurring in the 1335 high risk pre- and postmenopausal patients. Numerical calculations are only provided for the censor and include methods. The data are grouped into intervals for the purposes of comparison. The estimates are Kaplan-Meier estimates. In this case, true censored observation in a specific interval are considered at risk of any event for the entire interval. The graphs presented in Figure 3 however, take into account the actual observed event times.

| *T* | *N* | *LR* | *OE* | *TC* | $p_{LR}$ | $p_{OT}$ | $S_{EFS}$ | $Q^{(Ce)}$ | $Q^{(In)}$ |
|---|---|---|---|---|---|---|---|---|---|
| 0 | 1335 | | | | | | 1.000 | 0.000 | 0.000 |
| 0–1 | 1335 | 44 | 99 | 14 | 0.0330 | 0.1071 | 0.893 | 0.033 | 0.033 |
| 1–2 | 1178 | 50 | 100 | 98 | 0.0424 | 0.1273 | 0.779 | 0.074 | 0.071 |
| 2–3 | 930 | 27 | 76 | 87 | 0.0290 | 0.1108 | 0.693 | 0.101 | 0.093 |
| 3–4 | 740 | 16 | 55 | 87 | 0.0216 | 0.0959 | 0.626 | 0.120 | 0.108 |
| 4–5 | 582 | 11 | 41 | 80 | 0.0189 | 0.0893 | 0.570 | 0.137 | 0.120 |
| 5–6 | 450 | 8 | 28 | 56 | 0.0178 | 0.0800 | 0.525 | 0.152 | 0.130 |
| 6–7 | 358 | 3 | 17 | 57 | 0.0084 | 0.0559 | 0.495 | 0.159 | 0.135 |
| 7–8 | 281 | 1 | 12 | 55 | 0.0036 | 0.0463 | 0.473 | 0.162 | 0.137 |
| 8–9 | 213 | 2 | 6 | 48 | 0.0094 | 0.0376 | 0.455 | 0.170 | 0.141 |
| 9–10 | 157 | 1 | 10 | 28 | 0.0064 | 0.0701 | 0.423 | 0.176 | 0.144 |

N: Number of patients at risk of any event at the start of time interval T. LR: Number of Local Recurrences observed in time interval T. OE: Number of Other Events (Metastases, 2nd primary malignancies, Contralateral breast, Death) observed in time interval T. TC: Number of True Censored Observations observed in time interval T. $p_{LR}$: Conditional probability of failure from local recurrence in time interval T. $p_{OE}$: Conditional probability of failure from other events in time interval T. $S_{EFS}$: Event-free survival at the end of time interval T. $Q^{(Ce)}$: Estimate of cumulative incidence of Local recurrence (Censor) at the end of time interval T. $Q^{(In)}$: Estimate of cumulative incidence of Local recurrence (Include) at the end of time interval T.